# How Does the Land Rental Market Participation Affect Household Efficiency? Evidence from Rural China

**DOI:** 10.3390/ijerph192316175

**Published:** 2022-12-02

**Authors:** Xiaolin Guo, Guanming Shi, Linyi Zheng, Wenrong Qian

**Affiliations:** 1 West Institute for Economic Research, Southwestern University of Finance and Economics, Chengdu 611130, China; 2 College of Agricultural & Life Sciences, University of Wisconsin-Madison, Madison, WI 53706, USA; 3 China Academy for Rural Development, Zhejiang University, Hangzhou 310058, China; 4 Center for the Study of Socialism with Chinese Characteristics, Zhejiang University, Hangzhou 310058, China

**Keywords:** land rental market, technical efficiency, allocative efficiency, farm size

## Abstract

The active land rental market affects inter-household efficiency. Using nationally representative household panel data in China from 2017 to 2019, we estimate rural household efficiency with consideration of diversification, and analyze the effect of land rental market participation on household efficiency using a control function approach. Results show that: (1) the mean technical efficiency and allocative efficiency were 0.45 and 0.64 in 2017 and 0.44 and 0.66 in 2019, respectively, suggesting the existence of efficiency loss; (2) land rented-in activities improve rural households’ technical efficiency but not allocative efficiency; (3) land rented out activities decrease household technical efficiency but increase allocative efficiency. Further analysis showed that farm size heterogeneity might be the main reason for the efficiency difference. Households with large farms and plot sizes on rented land have significant advantages in technical efficiency and insignificant impacts on allocative efficiency. The results explain why the land rental market promotes efficient land transfer, but households with a large farm size are still rare in rural areas. Finally, we provide suggestions on how to stimulate the land rental market under diversification circumstances.

## 1. Introduction

For developing countries, increasing rural household efficiency through the land rental market is essential to raise farm productivity and mitigate income disparity [1]. As land is one of the most important production factors for rural households, the land rental market makes it possible for small-scale farmers to expand their farm size to become more productive [2]. Several studies suggest that the land rental market may improve land productivity [3], agricultural output [4], and production efficiency [5,6]. Yet, some studies argue that land transfer would have a negative impact on technical efficiency [7] and the outflow of land harms agricultural operators’ interests [8]. These contrasting results suggest the importance of a further understanding of the relationship between the land rental market and household efficiency.

China started to promote an active land rental market as early as 1986. With the land rental market becoming more efficient, migrating households can easily liquidate their land to the remaining farm households and allocate more time to off-farm jobs [9]. Under this circumstance, rural households participate in various activities besides agriculture [10]. For those who are still engaged in agricultural production, these diversified activities could be deemed as a strategy to employ household resources to increase income sources. This diversification behavior is also common in other countries [11,12]. Within the diversification scope, an output-oriented DEA can be appropriate for farms that have multiple outputs and inputs in diversified activities [13]. The small household diversified behavior is common in China; however, the existing discussions from the agricultural side may limit the understanding of the land rental market on household efficiency [14].

Low household efficiency is another important issue in China. A strand of literature established discussions on the causes of low household efficiency. The relatively small and equally allocated farmland [15], the surplus of labor in the limited contracted land [16], and the transaction cost during the land rental process [7] are the main reasons discussed in the existing literature. However, what would be the effect of an active land rental market on the expansion of the farm size, and what would be the relationship between farm size and household efficiency? These are important questions to be analyzed and answered in this paper.

In light of the above, this research aims to assess the rural household efficiency with consideration of diversification in China to investigate how the land rental market affects household efficiency, and how the effect varies with different farm sizes. This study uses nationally representative household panel data and an output-based Data Envelopment Analysis (DEA) method to calculate household efficiency. First, we calculate the rural household efficiency with different components of agricultural and non-agricultural income included in the output variables to measure the households’ performance [14,17]. Second, the effect of land rental market participation on technical efficiency and allocation efficiency is estimated. Third, this paper further discusses the heterogeneity effect of different farm sizes on household efficiency. 

The rest of this paper is organized as follows: Section 2 describes the history of land rental market development in rural China, shows an intuitive case of the conceptual model, and provides the research hypotheses. Section 3 describes empirical methodology, the data, and variable selection. Section 4 provides the basic regression results, a robustness check, and two further discussions. Finally, Section 5 provides the conclusions and policy implications.

## 2. Background and Theory

Traditional small-scale farmers remain a fundamental part of China’s agricultural production since the implementation of the household responsibility system (HRS) [18]. This system, which came to replace two decades of collective farming, grants ownership to the village collective and entrusts individual households to make their production on long-term contracted land [19]. In the initial stage of HRS, the contract to the collective cannot be changed without authorization, and there are specific grain production requirements. Each farmer cultivates less than 2 mu (15 mu = one hectare) per capita in most cases [20]. This relatively low per capita land resource ignores the agricultural production efficiency of different households and results in production inefficiency [21]. 

To improve land transferability and improve agricultural labor productivity, the Chinese central government began to encourage a market-oriented land rental market as early as 1986. This encourages the concentration of cultivated land to efficient farmers to help them achieve an appropriate scale of farming. Farmers who cannot cultivate or participate in off-farm work can hand over their contracted land to the collective, or subcontract it by themselves. One thing that should be noticed is that, although the land could be subcontracted by farmers themselves, their crop species should still strictly follow the contract from the collective, and cannot be changed without authorization in this stage. In 1988, legal restrictions on land that cannot be leased or sold were relaxed. After 2000, rural land reform mainly intended to adapt and adjust the farmland contracted by the households with migrant workers. Subsequently, in 2003, the implementation of the Rural Land Contracting Law confirms the management right of rural households; rural households can independently carry out agricultural production and obtain benefits. Farmers can also receive income from land circulation even if they are no longer directly engaged in agricultural production on their contracted land. 

With the development of rural land reform, land plays a strategic role in rural areas and household income diversification behavior is more prominent [9,22]. In 2014, the Chinese central government proposed the “three rights (ownership, contract rights, management rights) separation” of contracted land to guide the orderly circulation of rural land management rights. Owing to the deepening land reform, the national agricultural land circulation rate increased from 26% in 2013 to 37% in 2017. 

**Figure 1:**
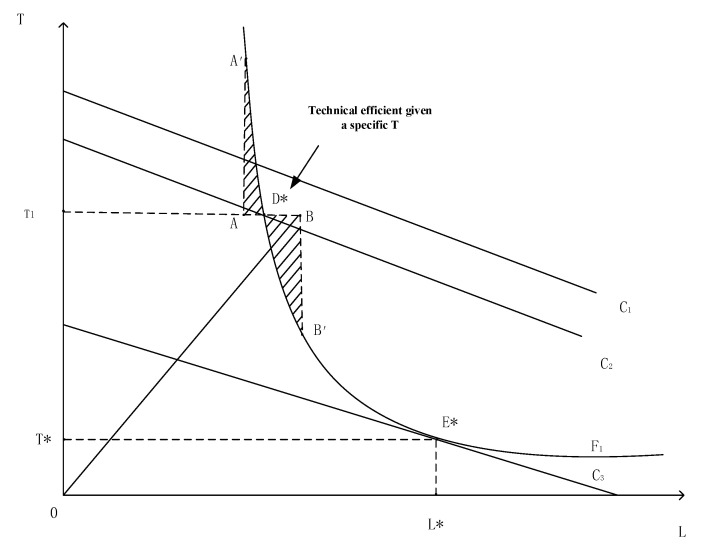
The two-factor technical efficiency and allocative efficiency.

To better understand the effect of household land rental market participation behavior on household efficiency, we show an intuitive and simple case. Assume a rural household generates a total income, Y, which consists of the agricultural income (p′q) and off-farm income (N), where ***q*** is a vector of the household’s agricultural output, and ***p*** is the exogenous output prices. To simplify the efficiency analysis, we consider a case of two inputs and a single output household production function in Figure 1. The two inputs are land (***L***) and other inputs (***T***). Other inputs include self-owned labor, hired labor, fertilizers, pesticides, and other agricultural inputs. In Figure 1, F1 is the targeted isoquant line, the C_1_, C_2_, and C_3_ are isocost lines. The slope of the isocost lines equals the ratio of the input prices of ***L*** and ***T***. We assume exogenous prices for both ***T*** and ***L***, thus the isocost lines are parallel in Figure 1.

All the points on F_1_ are technically efficient. Point E*, where the isocost line C_3_ and the isoquant F_1_ are tangent, denotes that ***L*** and ***T*** achieve both allocative and technical efficiency. In other words, households in E* make the best of resources from the cost perspective. Points A and B represent two households, each with initial land endowment and other inputs endowment. Conditional on a certain production constraint set, the T_1_D* represents the technical efficiency use of ***L*** under a given input ***T*** (OT_1_). In this case, TE can be simply defined as in Equation (1).

(1) $$TE\left(L,\;T,\;y,N,X\right)=Min_{\theta }\;\{\theta :\;\left(L,\;T;\;\left(p\prime q\right)/\theta ,N/\theta \right)\in X,\theta >0\},$$

where X is a certain production constraint set that the household faces. From Equation (1), TE=1 means that the household is engaged in agricultural production at the production frontier, which we call “technologically effective”. *TE* < 1 means that the technology is inefficient, where $\left[1–TE\right]$ represents the ratio of the distance to the technical effectiveness in the output.

We explain two examples in Figure 1 to illustrate how the land rental market participation would affect household efficiency. Point A in Figure 1 represents one type of inefficient household. Without the land rental market, the household in point A could only adjust their other endowment ***L*** to point A′ to achieve technical efficiency. If they could rent in land to increase ***T***, they could get to the technical efficiency point D* quicker by land rental market participation than without participation. The points in the upper dashed area are all in a similar case. If the land rented in behavior almost does not move the point to a lower isocost line, it will not affect allocative efficiency much. The allocative efficiency, AE, can be simply defined as follows:

(2) $$\;AE\left(p,T,L,x,H,X\right)=\left[p^{\prime }\;\left(y/TE\right)+N/TE\right]/R\left(p,T,L,x,H,X\right),$$

where $\left(y/TE,\;N/TE\right)\;$ is the technical validity variable in Equation (1); 0≤AE≤1. It implies the profit maximization at a given price based on multiple inputs. Therefore, we propose the first hypothesis.

**Hypothesis** **1:** *Land rented in increases rural household’s technical efficiency, but it has less impact on allocative efficiency.*

Point B in Figure 1 is the other case. Rational households in B could improve their efficiency in two ways. One is to rent out land and achieve technical efficiency in D*. In this case, the household would improve its technical efficiency but have little impact on allocative efficiency, such as in the former case. However, if the rational households adjusted their other endowment besides land; for example, from the point B to B′, the household could get easier to achieve allocative efficiency, though have less impact or negative effects on the technical efficiency. The points in the lower dashed area are in this similar case. Therefore, the second hypothesis is proposed as follows:

**Hypothesis** **2:** *Land rented out would have less effect on technical efficiency and it would have a positive effect on allocative efficiency.*

## 3. Data and Method

### 3.1. Data Source

The data used in this study were from the 2017 and 2019 China Rural Household Panel Survey (CRHPS) collected by Zhejiang University. The survey contains comprehensive information on rural households in China, including basic demographics, land use and circulation, agricultural and non-agricultural production and operation, migration, and more. The calculation of household efficiency needs detailed agricultural input and output information, which are not available until 2017, so we only use the 2017 and 2019 sample data. This study focuses on the subsample of households that contracted land and/or households that originally had contracted land and rented out their land.

Taking into account regional differences in crop species and the price differences of crop varieties [23], this study divides the sample into five groups based on their staple grain crops, derived from the *China Rural Statistical Yearbook*. Moreover, this study calculates the real values of the expenditures on fertilizers, pesticides, seeds, agricultural employment, machinery (machinery rental and small farm machinery), and other agricultural production costs and outputs. At the same time, since DEA is sensitive to outliers, the data were trimmed at 5% for outliers (e.g., under the current technology condition, abnormal yields of rice, wheat, maize, potatoes, and sweet potatoes are defined as 1000, 800, 1500, 5000, and 4000 kg/mu, respectively). Finally, 15,347 households in 159 prefecture-level cities in 22 provinces (autonomous regions or municipalities) from 2017 to 2019 were identified. The numbers of observations from the different regions are shown in Table 1.

### 3.2. Empirical Methodology

The traditional production function using the parametric method, DEA, and stochastic frontier analysis (SFA) are the three main methods for calculating production efficiency. In this paper, we use the DEA method for the following two reasons: First, compared with traditional production functions, DEA (and SFA) can further decompose efficiency into separate components [24]. Second, since agricultural production is a complex process, compared with SFA, DEA can avoid model selection bias because it does not need to specify the setting of the model [25]. We implement an output-oriented DEA method proposed in [14] to estimate the TE and AE by using R on the remote platform provided by Zhejiang University (http://210.32.137.90/s/lib/libtb/show/1585 (accessed on 27 September 2022)). 

The input and output variables used in the efficiency analysis were chosen based on the existing literature. In view of the data availability, the input variables used in the efficiency analysis are actual cultivated land (defined as collectively allocated contracted land plus land rented in, and minus land rented out), the number of male family laborers over 15 years old, number of female laborers over 15 years old, hired labor costs (Yuan), chemical fertilizer expenditures (Yuan), pesticide expenditures (Yuan), seed expenditures (Yuan), rental machinery costs (Yuan), and other expenditures for agricultural production (Yuan). Two types of output values were considered in the DEA: agricultural income and off-farm income. To better reflect the different sources of agricultural output, we include different items, such as the output value of rice, wheat, maize, cash crops, and other crops (including the output value of forest plantations, aquaculture, other crops, and livestock). Off-farm income includes off-farm and land rental income. After the process of identifying and excluding the potential outliers mentioned in the data source part, detailed summary statistics of input and output variables are shown in Table 2.

Table 3 presents the results of TE and AE by regional and farm-size-based differences. The median amount by regional main crops shows that the technical efficiency loss is more prominent than the allocative efficiency loss. As the allocative efficiency measures households’ ability to maximize their profit using the optimal level of inputs, it shows that the household indeed allocates the household resources considering the price issue [26]. It also highlights the need to calculate the two types of rural household efficiency. In the rice production areas, the technical efficiency loss of farmers is the most obvious (around 60%), and the allocative efficiency is the highest among the five regions. For the other four regions, the mean value of technical and allocative efficiency is in a similar range. As for the efficiency difference among different farm sizes, we could easily get an illustrative result that the larger farm size benefits the technical efficiency, but also have a lesser or negative impact on allocative efficiency. The mean amount of difference in average plot size shows a similar result. What is more, the mean value of technical efficiency and allocative efficiency (around 0.45 and 0.65, respectively) shows that there is a large number of rural households experiencing efficiency loss in 2017 and 2019.

Since the panel data used in this paper are in two-year intervals, we assume that all farmers faced the same conditions of technological progress, which is also consistent with the conceptual model. Our measurement of TE and AE is output-based, which does not assume a perfect input market [13]. After obtaining the household technical efficiency and allocative efficiency, we used Equation (3) to analyze the effect of land transfer on rural household efficiency. All of the computational exercises except efficiency calculation were performed on the remote platform using Stata version 15.1.

(3) $$E_{ict}=\alpha +\beta LandT_{ict}+\theta X_{it}+\gamma D_{c}+\vartheta Z_{t}+v_{it}$$

where $E_{ict}$ is the technical efficiency or allocative efficiency of the *i*-th household in year *t*, city *c*. $LandT_{ict}$ is a binary variable indicating whether the household rented land or not (1 = land rented in/out; 0 = not participate in the land rental market) in year *t*; $X_{it}$ represents the control variables. In addition, we added prefecture-level city fixed effects $D_{c}\;$ and year fixed effect $Z_{t}\;$ to control for the variables that do not vary with year and city; $v_{it}$ represents the error term and β is the main parameter we are interested in. Given that the probability distribution of the dependent variable is more similar to a mixed distribution composed of a discrete point and a continuous distribution, the Tobit model can obtain a more consistent estimator compared with ordinary least squares (OLS). 

The study is to identify the causal effect of participation in land rental markets on rural household efficiency. First, confounders that affect both the land rental market participation decision and rural household efficiency would affect the identification. Second, there is an obvious reverse causality, which may cause endogenous problems in model estimation. Agricultural production efficiency may directly affect the decision-making of farmers’ families to participate in the land rental market, that is, whether the family participates in the land rental market is a “self-selection” and “non-random” behavior. With these considerations in mind, following the practices of [14,27], this study used a control function approach to alleviate potential endogeneity problems. In linear regressions, the control function method is essentially the same as the instrumental variable. Due to the main dependent variable being a restricted variable ranging from 0 to 1 in this study, the control function method is more effective for non-linear models with endogenous explanatory variables [28]. This study used the predicted probability of the household land rental participation as a control function of whether the household actually rented [3,29] in Equation (4).

(4) $$LandT_{ict}^{*}=\alpha +\delta OtherLand{T_{ratio}}_{it}+\theta X_{it}+\gamma D_{c}+\vartheta Z_{t}+v_{it},\;\left\{\begin{array}{c} LandT_{ict}=1\;,\mathrm{if}\;LandT_{ict}^{*}>0\\ LandT_{\mathrm{ict}}=0\;,\mathrm{Other} \end{array}\right.\;.$$

Specifically, the average rent-in and rent-out cultivated land area ratio of other villages within the same prefecture-level city, except for the village where the household is located ($OtherLand{T_{ratio}}_{i}$ in Equation (4)), was used as the control function, and δ is the coefficient of the control function. The land rental information of the other villages in the same city affects the household rent decisions, but this has no direct relationship with the output of their agricultural production and operation, because rational households make decisions mainly based on their own interests [30]. The tenure security level might be similar in the same region, but it is just a reference or a predicted value for whether the household participates in the land rental market [31]. Therefore, it is reasonable to use the predicted probability of household land rent decisions as the indicator of whether the household participates in the land rental market.

## 4. Results and Discussion

### 4.1. Description of Variables

The main dependent variables are the calculated household technical and allocative efficiencies in Table 4. Overall, from 2017 to 2019, the technical efficiency decreased from 0.45 to 0.44 (Table 4). This indicates that farmers could feasibly increase their output by 55% if they reach their best production condition. The average value of allocative efficiency shows a slight increase, from 0.64 to 0.66 between the two years. The main explanatory variable came from the CRHPS household questionnaire, which asked, “Whether your family rent in land or not last year” and “Whether your family rent out management rights of contracted rural land last year?” Between 2017 and 2019, the proportion of land rented in decreased from 8.5% to 7.3%, and the proportion of land rented out increased from 25.7% to 26.2%. Control variables that may affect farmland rental decisions and household efficiency were also included. 

The amount of contracted land per capita of the households increased slightly from 1.84 to 1.86 mu. The land for which land rights were confirmed and certificated increased dramatically from 65.1% to 77% from 2017 to 2019 [32]. The proportion of households with unpaid debt increased from 9.8% in 2017 to 11.5% in 2019. The proportion of farmers using machinery also increased, from 78.8% in 2017 to 82.5% in 2019. The logarithm of grain output per capita and the logarithm of grain output per mu both declined. The distance between the village and the town center to which it belongs was approximately 5 km.

### 4.2. The Effect of Land Rental Market on Rural Household Efficiency

The efficiency analysis in Section 3 highlights the fact that, to understand the effect of the land rental market on efficiency, both technical and allocative efficiency should be examined [33]. Table 5 shows a highly significant negative correlation between the control function “predicted household rent in/out rate” and the potential endogenous variable “whether the household participates in the land rental market”. A possible reason is that the farmland resources in the prefecture-level city are limited, and the land rental behavior of other villages crowds out the village farmland demand [34]. The results for the Hhlandin_cf and Hhlandoff_cf both show that the control functions included in the production efficiency equation are significant.

The results preliminarily verify research Hypothesis 1, that is, households that rented land achieved higher technical efficiency than households that did not, which is consistent with the literature that did not consider multiple outputs [35]. By employing the output-oriented DEA method, we could analyze the effect of allocative efficiency with consideration of household diversified income sources. We found that land rented in has no effect on allocative efficiency (column 2). Previous studies show that land rented in could improve agricultural productivity [36], and mainly discuss the improvement from technical efficiency [37]. We found that land rented out increases households’ allocative efficiency in column 4. Using cross-section data in 2009 in rural China, [14] finds participating in the land rental market improves allocative efficiency. Our analysis indicates that these improvements come from land leasing out. Furthermore, consistent with the second case when formulating Hypothesis 2, there is a significant negative effect on technical efficiency [38].

### 4.3. Robustness Check

We show that our main results in Table 5 are robust to alternative specification choices. Firstly, we exclude the households who transfer out their land entirely in the efficiency calculation and the basic regression. For such samples, it might have higher allocative efficiency and have low technical efficiency, thus affecting the marginal effect. Table 6 shows the robustness of the basic results.

Secondly, for the basic results in Table 5, we deem the households who rent in land and rent out land do not have systematic differences. In Table 7, we change the control group and do not include the opposite part in the regression. Households who rented out land are excluded from the control group of the land rented in column 1 and 2, the same as the households who rented in land in column 3 and column 4. The marginal effects of the impact of the land rental market on rural household efficiency are similar but slightly larger than the basic results. It verifies that it is feasible to both include land rent in and rent out into the regression. The marginal effect in Table 6 and Table 7 shows that we estimate the lower bound and underestimate the effects of the land rental market.

Next, we estimate the effect of the land rental market by regions to test the regional heterogeneity in Table 8. The impact of farmland rented in on the technical efficiency in regions 2 (Henan, Shandong, Shanxi, Shaanxi, Gansu, and Hebei), and 4 (Heilongjiang, Jilin, and Liaoning) are greater than the average treatment effects (0.227) in Table 5. This study does not find a significant effect of land rented in on the technical efficiency in regions 1 (Anhui, Hubei, Jiangsu, Sichuan, and Yunnan), 3 (Hunan, Guangxi, Guizhou, and Chongqing), and 5 (Fujian, Guangdong, Zhejiang, and Jiangxi). It indicates that the impact of farmland rented in on technical efficiency mainly comes from traditional farming areas in China. In addition, the northeastern region (region 4) does not show a significant impact on allocative efficiency after the land is rented out, and the other four regions all have a significant improvement in allocative efficiency after the land is rented out. These results indicate that the effect of the land rental market on agricultural household efficiency has significant regional heterogeneity [39].

### 4.4. Further Discussion

Although the land rental market is active, the household relative efficiency is still relatively low in rural China. Our findings in Table 4 show that the mean technical efficiency is around 0.45 in the research period, suggesting the existence of a large number of rural households experiencing efficiency loss in rural China. Some studies show that the relatively small farm size is the main driving force of the low household efficiency during the development process [40]. We then further explore whether land rented in improves technical efficiency through larger farm size. 

Table 9 explains the different effects of land rented in by farm size. According to the first two columns in panel A, we find a significant positive effect between farm size and technical efficiency. These results indicate that the expanding management of scale benefits technical efficiency improvement [41]. Some other studies using medium- and large-scale farm level data find an inverse relationship between farm size and technical efficiency [42,43]. As shown in Table 3, the average land per capita is only 1.85 in our research, thus we did not find a further inverse relationship.

As far as we know, limited literature analyzes the relationship between farm size and household allocative efficiency. However, allocative efficiency is beneficial to understand the household resource allocation effect, especially in diversification cases. Column 3 in panel A shows the significant negative effect of land rented in on household allocative efficiency. It indicates the small farm size prevents the small household from achieving higher allocative efficiency [44]. Column 4 shows that even if households expand their arable area, there is still no significant effect on allocative efficiency improvement. This suggests that agricultural production is relatively low in efficiency when income is considered in the current stage. Other studies examining the effect of land rentals on farm household income find similar results [29]. However, the household income could not give us information on household efficiency status. Panel B shows similar results when we change the measurement of farm size to the average plot size. 

In this section, we obtain new sights into farm size heterogeneity to explore the relationship between the land rental market and household efficiency. According to the results in Table 9, we find that the expansion of farm size may not necessarily improve allocative efficiency when accounting for the respective prices of input. It goes some way to explain why households with large farm sizes are still rare in China.

## 5. Conclusions 

Using household panel data, this study investigated the efficiency of rural households in China in the years 2017 and 2019. We then used the calculated efficiency to explore the effect of land rental market participation on it and discuss the farm size heterogeneity. Compared to existing research, this study focused on household efficiency with consideration of diversification. 

Our analysis shows that the mean technical efficiency is around 0.45 in the research period, and the mean allocative efficiency is around 0.65, both suggesting there is much room for improving the performance of agricultural households. After correcting for potential endogeneity issues, this study finds that land rented in increased the technical efficiency of households by 22.7% in the research period. The further detailed results show that more arable land benefits households’ technical efficiency, but do not affect allocative efficiency. The results suggest that scale farming is indeed necessary to improve agricultural production, but has limited impact on household profit maximization behavior and income growth. Policies to stimulate household efficiency should contribute to improving agricultural technology to adapt to the existing land conditions. The government could provide subsidies on agricultural input, such as agricultural machinery and tool subsidies, to those who transfer in land and operate a large farm. The results also show that land rented out decreases household technical efficiency. The households that still hold land after part of their land is rented out experienced large negative effects on technical efficiency. This is largely due to the decrease in farming sizes. 

The different signs of the effects of the land rental market on technical and allocative efficiency manifest the importance of research on efficiency from different dimensions. Although this study focused exclusively on China, our approach could be extended to other countries in which household work on diversified activities is utilized to maximize their income. This study also has some limitations. First, in principle, it would be desirable to use household plot-level-specific input and output information to calculate household efficiency, but data constraints bind. Second, for households using various inputs beyond land and physical agricultural resources, such as human capital, the application of output-oriented calculation of household efficiency may be cautious. 

Faced with those opportunities, the literature can help explain the incomplete and low efficiency of the land rental market in rural China. Future research can enrich the literature by analyzing the characteristic of households in different land rental statuses. Additionally, with institutional barriers reduced and technology improved, there will be more opportunities for further land rental market development and household efficiency improvement.

## Figures and Tables

**Table 1:** Classification of Regions by their Main Staple Crops.

Region	Province	Crops	Number of Observations		
			All	2017	2019
1	Anhui, Hubei, Jiangsu, Sichuan, and Yunnan	Rice, wheat, and maize	3768	1848	1920
2	Henan, Shandong, Shanxi, Shaanxi, Gansu, and Hebei	Wheat and maize	4464	2354	2110
3	Hunan, Guangxi, Guizhou, and Chongqing	Rice and maize	2565	1246	1319
4	Heilongjiang, Jilin, and Liaoning	Maize	1963	1133	830
5	Fujian, Guangdong, Zhejiang, and Jiangxi	Rice	2587	1432	1155
Total			15,347	8013	7334

**Table 2:** Summary statistics of inputs and outputs of rural household.

	2017		2019	
Abbreviations of the Variables (Unit)	Mean	S.D.	Mean	S.D.
**Output variables (Yuan)**				
Output value of rice	1092.87	4512.72	934.44	3493.00
Output value of wheat	619.63	1605.52	508.09	1448.98
Output value of maize	2805.27	8053.08	2059.90	6337.23
Output value of cash crops	902.60	2963.76	1129.48	3695.71
Output value of other crops	561.58	1931.68	509.39	1766.72
Off-farm income (Yuan)	32,295.21	36,935.49	37,949.56	39,907.77
**Input variables (Yuan)**				
Cultivated land (mu) (Yuan)	5.02	9.26	4.67	7.52
Other type agricultural land (mu)	0.47	1.80	0.847	3.14
No. of male family laborers over 15 years old	1.37	0.72	1.307	0.692
No. of female laborers over 15 years old	1.32	0.70	1.24	0.68
Hired labor costs (Yuan)	49.27	308.72	51.45	337.66
Chemical fertilizer expenditures (Yuan)	805.11	1398.57	775.55	1336.94
Pesticide expenditures (Yuan)	281.85	431.62	212.90	403.60
Seeds costs (Yuan)	321.51	657.55	318.43	646.09
Rental machinery costs (Yuan)	388.44	826.36	351.02	725.98
Other expenditures for agricultural production (Yuan)	241.85	597.58	221.43	549.60

Notes: S.D. is the abbreviation for standard deviation. Output value of other crops includes the output value of forest plantations, aquaculture, other crops, and livestock. Off-farm income includes off-farm and land rental income. All monetary inputs and outputs are expressed in RMB and deflated with reference to year 2015.

**Table 3:** Summary statistics of rural household efficiency.

		TE		AE	
Groups	Number of Observations	Mean	S.D.	Mean	S.D.
**A: By Regional Main Crops**					
Rice, wheat, and maize	3768	0.455	0.298	0.621	0.367
Wheat and maize	4464	0.467	0.293	0.617	0.355
Rice and maize	2565	0.482	0.304	0.603	0.371
Maize	1963	0.41	0.31	0.674	0.191
Rice	2587	0.395	0.295	0.764	0.35
**B: By Farm Size**					
Less than 5 mu	10,535	0.391	0.271	0.685	0.346
More than 5 mu	4812	0.570	0.323	0.568	0.338
**C: By Average Plot Size**					
Less than 1 mu	9186	0.405	0.285	0.673	0.352
More than 1 mu	5482	0.521	0.311	0.596	0.335
**Total**	15,347	0.447	0.300	0.648	0.348

Notes: S.D. is the abbreviation for Standard deviation.

**Table 4:** Variable selection and summary statistics.

		2017		2019	
Abbreviations of the Variables	Definition of the Variables (Unit)	Mean	S.D.	Mean	S.D.
**Rural household efficiency**					
TE	Technical efficiency	0.450	0.301	0.444	0.300
AE	Allocative efficiency	0.641	0.353	0.655	0.342
**Land rental characteristics**					
Landin	Rent in (1 = yes; 0 = no)	0.085	0.279	0.073	0.260
Landoff	Rent out (1 = yes; 0 = no)	0.257	0.437	0.262	0.440
**Socio-economic characteristics**					
Headgender	Gender of household head (1 = male; 0 = female)	0.893	0.310	0.845	0.362
Headage	Age of household head	53.488	11.232	54.295	10.521
Headedu	Educational attainment of household head	2.699	1.075	2.714	1.025
Familysize	Number of family members in the household	3.772	1.624	3.615	1.618
Female_ratio	Proportion of female laborers in the household	0.366	0.174	0.362	0.182
Up_64	Proportion of family members above 64 years old in the household	0.426	0.670	0.443	0.681
Juniorhigh_ratio	Proportion of educational attainment above high school in the household	0.364	0.336	0.376	0.348
Child_adult	Proportion of children in the family (%)	0.210	0.309	0.207	0.328
Migrant	Migrant off-farm laborers in the household (1 = yes; 0 = no)	0.239	0.426	0.155	0.362
Land_percapita	Amount of contracted land per capita in the household (mu)	1.836	3.017	1.855	2.607
Land_cer	Land confirmation and certification (1 = yes; 0 = no)	0.651	0.477	0.770	0.421
Debt	Household has unpaid debt (1 = yes; 0 = no)	0.098	0.297	0.115	0.319
Machine	Use of machinery (1 = yes; 0 = no)	0.788	0.409	0.825	0.380
Migtotal_ratio	Proportion of migrant off-farm laborers in the household	0.089	0.179	0.057	0.150
Migprov_ratio	Proportion of out-province migrant, off-farm laborers in the household	0.032	0.107	0.023	0.089
Migcity_ratio	Proportion of out-city migrant, off-farm laborers in the household	0.048	0.131	0.033	0.109
**Village-level characteristics**					
Othervillandin_ratio	Proportion of the amount of farmland rented in out of the total farmland in the city except this village	0.070	0.068	0.061	0.062
Othervillandoff_ratio	Proportion of the amount of farmland rented out of the total farmland in the city except this village	0.222	0.172	0.224	0.177
Inmigranthhs_ratio	Proportion of in-village migrant in the city except this village	0.044	0.057	0.043	0.092
Dist	Distance of village to the town center (km)	4.984	5.947	4.400	5.480

Notes: S.D. is the abbreviation for Standard deviation. The educational attainment of the household head is a categorical variable: illiteracy = 1; elementary school = 2; junior high school = 3; high school = 4; technical secondary school/vocational high school = 5; junior college/ higher vocational college = 6; undergraduate degree = 7; master’s degree = 8; and doctoral degree = 9.

**Table 5:** Land transfer and rural household efficiency: control function results.

	(1)	(2)	(3)	(4)
	TE	AE	TE	AE
	Tobit	Tobit	Tobit	Tobit
Landin	0.227 *** (−0.053)	−0.013 (−0.051)		
Landoff			−0.165 *** (−0.022)	0.196 *** (−0.021)
Hhlandin_cf	−0.055 ** (−0.025)	−0.024 ** (−0.011)		
Hhlandoff_cf			0.039 *** (−0.012)	−0.030 *** (−0.011)
Controls	YES	YES	YES	YES
City FE	YES	YES	YES	YES
Year FE	YES	YES	YES	YES
N	15,347	15,347	15,347	15,347

Notes: The marginal effects of the Tobit and Probit models are reported; *** and ** denote significance at the 1% and 5% levels, respectively. Standard errors (in parentheses) are clustered at the city level. Regarding the joint significance statistics, the model is globally significant (Prob > F is 0.0000).

**Table 6:** Land transfer and rural Household efficiency: changing the samples of dependent variables.

	(1)	(2)	(3)	(4)
	TE	AE	TE	AE
	Tobit	Tobit	Tobit	Tobit
Landin	0.284 *** (0.055)	−0.062 (0.252)		
Landoff			−0.241 *** (0.032)	0.140 *** (0.031)
Hhlandin_cf	−0.089 *** (0.027)	0.067 ** (0.027)		
Hhlandoff_cf			0.083 *** (0.016)	−0.015 *** (0.003)
Controls	YES	YES	YES	YES
City FE	YES	YES	YES	YES
Year FE	YES	YES	YES	YES
N	13,180	13,180	13,180	13,180

Notes: The marginal effects of the Tobit and Probit models are reported; *** and ** denote significance at the 1% and 5% levels, respectively. Standard errors (in parentheses) are clustered at the city level. Regarding the joint significance statistics, the model is globally significant (Prob > F is 0.0000).

**Table 7:** Changing the control group of the key independent variables.

	(1)	(2)	(3)	(4)
	TE	AE	TE	AE
	Tobit	Tobit	Tobit	Tobit
Landin2	0.266 *** (−0.059)	−0.037 (−0.057)		
Landoff2			−0.152 *** (−0.023)	0.204 *** (−0.022)
Hhlandin_cf	−0.089 *** (−0.030)			
Hhlandoff_cf				−0.042 *** (−0.012)
Controls	YES			YES
City FE	YES			YES
Year FE	YES			YES
N	11,366	11,366	14,130	14,130

Notes: The marginal effects of the Tobit and Probit models are reported; *** denotes significance at the 1% levels. Standard errors (in parentheses) are clustered at the city level. Prob > F is 0.0000 in all models.

**Table 8:** The effect of land transfer on rural household efficiency by region.

	Region 1	Region 2	Region 3	Region 4	Region 5
**Panel A**	Technical Efficiency				
Landin	0.0467 −0.104	0.406 *** −0.1345	0.135 −0.1482	0.371 *** −0.1141	0.105 −0.1517
**Panel B**	Allocative Efficiency				
Landin	−0.00247 −0.101	0.00494 −0.1342	−0.174 −0.1451	0.0947 −0.0777	−0.330 ** −0.143
**Panel C**	Technical Efficiency				
Landoff	−0.206 *** −0.0517	−0.213 *** −0.0504	−0.203 *** −0.0753	−0.180 ** −0.071	−0.141 ** −0.0572
**Panel D**	Allocative Efficiency				
Landoff	0.177 *** −0.0545	0.190 *** −0.0548	0.443 *** −0.0825	−0.0135 −0.0436	0.385 *** −0.0778
N	3768	4464	2565	1963	2587

Notes: The marginal effects of the Tobit models are reported; *** and ** denote significance at the 1% and 5% levels, respectively. Standard errors (in parentheses) are clustered at the city level. Prob > F is 0.0000 in all the Tobit models.

**Table 9:** Land rented in, the effect of farm size on household efficiency.

	TE		AE	
**Panel A**	**Small Farm Size**	**Large Farm Size**	**Small Farm Size**	**Large Farm Size**
Landin	0.139 * (0.075)	0.235 *** (0.078)	−0.201 *** (0.076)	0.042 (0.073)
N	10,535	4812	10,535	4812
**Panel B**	**Small Average Plot Size**	**Large Average Plot Size**	**Small Average Plot Size**	**Large Average Plot Size**
Landin	0.128 (0.125)	0.343 *** (0.081)	−0.147 * (0.082)	0.073 (0.075)
N	9186	5482	9186	5482

Notes: The small farm size in this paper is defined as the household actual farming land is below 5 mu. The large farm size is defined as 5 mu or more. The average plot size is calculated by the actual farming size divides the plot number. The small average plot size is defined as 1 mu or less. The large average plot size is defined as more than 1 mu. The marginal effects of the Tobit models are reported; *** and * denote significance at the 1% and 10% levels, respectively. Standard errors (in parentheses) are clustered at the city level. Prob > F is 0.0000 in all the Tobit models.

## Data Availability

The data are not publicly available due to personal privacy and non-open access to the research program. The related code is available on request from the corresponding author.
